# Measurement of a True $\dot{\text{V}}$O_2max_ during a Ramp Incremental Test Is Not Confirmed by a Verification Phase

**DOI:** 10.3389/fphys.2018.00143

**Published:** 2018-02-27

**Authors:** Juan M. Murias, Silvia Pogliaghi, Donald H. Paterson

**Affiliations:** ^1^Faculty of Kinesiology, University of Calgary, Calgary, AB, Canada; ^2^Department of Neurosciences, Biomedicine and Movement Sciences, University of Verona, Verona, Italy; ^3^School of Kinesiology, The University of Western Ontario, London, ON, Canada

**Keywords:** maximal oxygen uptake, oxidative metabolism, exercise testing, aerobic performance, aging

## Abstract

The accuracy of an exhaustive ramp incremental (RI) test to determine maximal oxygen uptake ($\dot{\text{V}}$O_2max_) was recently questioned and the utilization of a verification phase proposed as a gold standard. This study compared the oxygen uptake ($\dot{\text{V}}$O_2_) during a RI test to that obtained during a verification phase aimed to confirm attainment of $\dot{\text{V}}$O_2max_. Sixty-one healthy males [31 older (O) 65 ± 5 yrs; 30 younger (Y) 25 ± 4 yrs] performed a RI test (15–20 W/min for O and 25 W/min for Y). At the end of the RI test, a 5-min recovery period was followed by a verification phase of constant load cycling to fatigue at either 85% (*n* = 16) or 105% (*n* = 45) of the peak power output obtained from the RI test. The highest $\dot{\text{V}}$O_2_ after the RI test (39.8 ± 11.5 mL·kg^−1^·min^−1^) and the verification phase (40.1 ± 11.2 mL·kg^−1^·min^−1^) were not different (*p* = 0.33) and they were highly correlated (*r* = 0.99; *p* < 0.01). This response was not affected by age or intensity of the verification phase. The Bland-Altman analysis revealed a very small absolute bias (−0.25 mL·kg^−1^·min^−1^, not different from 0) and a precision of ±1.56 mL·kg^−1^·min^−1^ between measures. This study indicated that a verification phase does not highlight an under-estimation of $\dot{\text{V}}$O_2max_ derived from a RI test, in a large and heterogeneous group of healthy younger and older men naïve to laboratory testing procedures. Moreover, only minor within-individual differences were observed between the maximal $\dot{\text{V}}$O_2_ elicited during the RI and the verification phase. Thus a verification phase does not add any validation of the determination of a $\dot{\text{V}}$O_2max_. Therefore, the recommendation that a verification phase should become a gold standard procedure, although initially appealing, is not supported by the experimental data.

## Introduction

Exercise physiologists have been interested in the measurements of oxygen uptake ($\dot{\text{V}}$O_2_) and maximal $\dot{\text{V}}$O_2_ ($\dot{\text{V}}$O_2max_) since early in the 20th century (Krogh and Lindhard, 1913; Hill and Lupton, 1923). Given the importance of $\dot{\text{V}}$O_2max_ as an integrative measure of different components of the cardiovascular and neuromuscular system to exercise, its measurement has been widespread not only as an indicator of human performance (Hoppeler and Weibel, 2000; di Prampero, 2003; Levine, 2008), but also as a marker of overall cardiovascular and cardiorespiratory function in different healthy and clinical populations (Frontera et al., 1990; Borrelli et al., 2003; Pogliaghi et al., 2006; Levine, 2008; Paterson and Warburton, 2010; Jensen et al., 2016) and different environmental conditions (Bringard et al., 2010; Doria et al., 2011). Similarly, measures of $\dot{\text{V}}$O_2max_ have been used to determine the efficacy of different exercise training interventions aimed at improving physiological function both from performance as well as health perspectives (Pogliaghi et al., 2006; Murias et al., 2010a; Bruseghini et al., 2015). Considering the importance of $\dot{\text{V}}$O_2max_ for assessment of performance and health, adequate protocols capable of establishing a true maximal value are needed to provide confidence for evaluation and longitudinal follow-up.

Early studies measuring expired gases and volumes from a Douglas bag promoted the use of testing protocols that required prolonged steps of progressively increased intensity; such protocols aimed to establish a steady-state or sustainable response in each step, until critical intensities of exercise prevented further steps from being completed (ÅSTRAND, 1960; Glassford et al., 1965). Under those conditions, a work rate (or speed) of exercise that resulted in no further increase in $\dot{\text{V}}$O_2_ despite the increase in energy demand was established and this plateau response was considered a true $\dot{\text{V}}$O_2max_, as proposed by Taylor et al. (1955). With the development of fast-response breath-by-breath gas exchange and volume analyzers, researchers started to move away from these types of time-consuming protocols (Buchfuhrer et al., 1983), and step incremental and ramp incremental (RI) tests became widely used to measure the $\dot{\text{V}}$O_2max_ response during exercise to exhaustion. RI protocols allow for rapid determination of $\dot{\text{V}}$O_2max_ as well as other valuable indexes such as the exercise intensity thresholds; however, some researchers have questioned the accuracy of RI tests to consistently provide a true $\dot{\text{V}}$O_2max_ value as a plateau in $\dot{\text{V}}$O_2_ is not always (or seldom) observed (Rossiter et al., 2006; Poole et al., 2008; Poole and Jones, 2017). To circumvent this limitation, secondary criteria such as an increase in blood lactate concentration [La] above 8 mmol·L^−1^, a heart rate response within 10 beats per minute (bpm) of the maximal predicted value, a respiratory exchange ratio (RER) higher than 1.10, and a rating of perceived exertion (RPE) >18, are commonly used to establish the attainment of a true $\dot{\text{V}}$O_2max_ response (ACSM, 2003). However, as indicated by Midgley et al. (2007) and Poole et al. (2008), secondary criteria do not seem valid for accurate determination of the attainment of $\dot{\text{V}}$O_2max_.

Another approach to establish the attainment of $\dot{\text{V}}$O_2max_ consists of executing a verification (or validation) phase subsequent to the RI test (Day et al., 2003; Midgley and Carroll, 2009). Although the exact origin of this model is not clearly established (Midgley and Carroll, 2009), Rossiter et al. (2006) proposed that, following a recovery period of five minutes subsequent to the RI test, a constant load exercise to exhaustion should be performed at either 95 or 105% of the peak power output (PO) obtained during the RI test. The use of a verification phase to confirm the attainment of a true maximum during a RI test in which a plateau in $\dot{\text{V}}$O_2_ may not be present, is based on the following assumptions:

(i) a RI test may fail to provide a true $\dot{\text{V}}$O_2max_; (ii) an upper limit to $\dot{\text{V}}$O_2_ exists and this value can only be underestimated but not overestimated; (iii) during a constant load exercise to the limit of tolerance in the very heavy/severe domain, $\dot{\text{V}}$O_2_ will project to $\dot{\text{V}}$O_2max_ within the time of task failure. Therefore, according to Rossiter et al. (2006), the lack of significant differences in the $\dot{\text{V}}$O_2_ response of the constant load verification phase, both at 95 and 105% of peak PO, compared to the highest $\dot{\text{V}}$O_2_ observed during the RI test, would provide the objective “plateau criterion” that often remains elusive during a RI test and confirm that a maximal value was obtained. Importantly, this protocol not only corroborated the presence of an upper limit of the $\dot{\text{V}}$O_2_ response, but it also indicated that the highest $\dot{\text{V}}$O_2_ values obtained during a RI were not different from those observed during the verification phase and likely reflected the achievement of a true $\dot{\text{V}}$O_2max_.

Recently, a review by Poole and Jones (2017) proposed the idea that the denomination of “peak” $\dot{\text{V}}$O_2_ ($\dot{\text{V}}$O_2peak_) is no longer acceptable and that validated $\dot{\text{V}}$O_2max_ results, as derived from a verification phase, should be presented in all future studies. An important point raised by Poole and Jones (2017) is that even though the RI test might yield a highly reproducible $\dot{\text{V}}$O_2max_ response in active or trained participants who are accustomed to pushing themselves to exhaustion, this may not be the case with less experienced, unmotivated, and/or clinical populations. Thus, even though a RI test might provide a trustable measure of $\dot{\text{V}}$O_2max_ in trained individuals, a verification phase should always be performed in any population unaccustomed to pushing to the upper limits of tolerance.

Although the verification phase appears appealing in providing a strategy to overcome the ongoing $\dot{\text{V}}$O_2_ max-peak debate, data from different studies do not convincingly support the usefulness or necessity of a verification phase either in the general healthy population or in specific clinical/frail/unfit populations. In sedentary (Astorino et al., 2009), recreationally active (Sedgeman et al., 2013; Nolan et al., 2014), overweight and obese (Wood et al., 2010; Sawyer et al., 2015) adults, as well as children (Barker et al., 2011), the $\dot{\text{V}}$O_2_ response during the verification phase was not higher than that observed during the RI test. This was also the case in a group of chronic heart failure patients (Bowen et al., 2012). These data could suggest that the verification phase provided a confirmation that a true $\dot{\text{V}}$O_2max_ was established in these subjects. However, these very same findings could just as plausibly indicate that the RI test *per-se* was an adequate protocol, yielding a true maximal response in most participants. Moreover, a recent study has shown that a verification phase resulted in a lower $\dot{\text{V}}$O_2_ value as compared to that observed during a graded incremental test (McGawley, 2017). Importantly, the possible usefulness of a verification phase has only been investigated in relatively small and homogeneous samples and (with the exception of McGawley (2017)) with a suboptimal statistical approach (i.e., only comparison of mean values). Additionally, healthy older adults, for whom $\dot{\text{V}}$O_2max_ is the strongest predictor of independent living (Paterson and Warburton, 2010) and for whom a verification phase would theoretically be needed for accurate $\dot{\text{V}}$O_2max_ determination (Poole and Jones, 2017), have never previously been evaluated. Establishing if a verification phase actually adds confidence in the achievement of $\dot{\text{V}}$O_2max_ in this population would contribute to further support or oppose the recommendation of such practice.

Poole and Jones (2017) recommended that the verification phase should be performed at intensities exceeding the peak PO obtained at the end of the RI test (e.g., 105% of peak PO). This is proposed in order to satisfy the plateau criterion (i.e., no further increase in $\dot{\text{V}}$O_2_ despite the increase in PO) that is the foundation of the $\dot{\text{V}}$O_2max_ concept. However, it should be noted that, due to the presence of the so called slow component of $\dot{\text{V}}$O_2_, any constant load PO above critical power will result in the achievement of $\dot{\text{V}}$O_2max_, provided that time to task failure is sufficiently prolonged (Poole and Jones, 2012). This well-known physiological phenomenon provides the rationale for using workloads that are not only above but also below maximal PO for the verification phase (Rossiter et al., 2006), with lower intensities possibly favoring compliance and reducing the risk associated to supramaximal exercise in older or clinical populations. Indeed, verification phases conducted at a submaximal PO yield $\dot{\text{V}}$O_2max_ values not different from those obtained from RI tests (Rossiter et al., 2006; Sedgeman et al., 2013).

Thus, given the uncertainties related to the ability of establishing $\dot{\text{V}}$O_2max_ during a RI test, and the proposal that a verification phase with constant load might confirm the RI $\dot{\text{V}}$O_2_ or identify that the RI test was not maximal, the goals of this study were to: (1) determine whether a constant load verification phase yields a $\dot{\text{V}}$O_2_ higher than the RI test; (2) examine the variation of the differences around the mean between the highest $\dot{\text{V}}$O_2_ of the RI test and the verification phase; (3) determine the role of age group (i.e., younger vs. older adults) on the possible difference in the $\dot{\text{V}}$O_2_ value between the RI test and the verification phase; (4) determine the role of the intensity of the verification phase (i.e., above or below the PO elicited at the end of the RI test) on the possible difference between the $\dot{\text{V}}$O_2_ value during the RI test and the verification phase. We tested the hypothesis that a verification phase (either below or above the end-RI intensity) would produce a higher $\dot{\text{V}}$O_2_ than that observed during the RI test.

## Materials and methods

This study combines sets of data collected in two different locations: The University of Western Ontario and the University of Verona. Although part of the $\dot{\text{V}}$O_2max_ data have been reported elsewhere (Murias et al., 2010b, 2011; Bruseghini et al., 2015; Keir et al., 2015) this is the first time that the $\dot{\text{V}}$O_2_ data derived from the RI test and verification phase have been reported together and compared.

### Participants

Data from a total of 61 healthy males (30 younger, Y: 25 ± 4 yr; 178 ± 6 cm; 79 ± 13 kg and 31 older adults O: 68 ± 5 yr; 174 ± 8 cm; 78 ± 10 kg; mean ± *SD*) were included in the present analysis. Eight younger and eight older adults performed the testing procedures at The University of Western Ontario (RI test + verification phase at 85% of peak PO at the end of the RI test). The remaining 22 younger and 23 older adults were tested at the University of Verona (RI test + verification phase at 105% of peak PO at the end of the RI test). All participants were volunteers and provided written informed consent to participate in the study. Participants were included in this database if they were aged between 18 and 90 years old, had performed a RI test and a subsequent verification phase, were relatively naïve to laboratory testing procedures (i.e., had not been involved in laboratory testing in at least the previous 12 months). All participants were recreationally active, community dwelling individuals, none of whom was involved in an organized endurance training regime when the original testing took place. Common exclusion criteria were obesity (body mass index > 30 kg/m^2^), smoking, involvement in any endurance training program within the previous twelve months, taking medications that would affect the cardiorespiratory or hemodynamic responses to exercise, or a history of cardiovascular, respiratory or musculoskeletal diseases. All subjects completed a PAR-Q+ questionnaire prior to enrolment and all older adults were medically screened by a physician before being accepted into the study. All procedures were approved by The University of Western Ontario Research Ethics Board for Health Sciences Research Involving Human Subjects or by the Ethics Board of the Department of Neurosciences, Biomedicine and Movement Sciences at the University of Verona.

### Protocol

Participants performed a maximal RI test from a baseline of 20 W to the PO that elicited exhaustion using increments of 25 W/min (Y) and 15–20 W/min (O). Based on the anticipated fitness level of O (Pogliaghi et al., 2014) a ramp slope (i.e., W/min increment) was chosen that would bring the participants to exhaustion within 10–12 min based on the ACSM (2003) guidelines. The RI test was performed on a cycle ergometer (Lode Corival 400; Lode B.V., Groningen, Holland) and $\dot{\text{V}}$O_2_ was measured throughout the test. After completion of the RI test, participants returned to cycling at the baseline PO of 20 W for 5 min, after which an instantaneous step increase in the PO was applied and the participants performed a constant load cycling exercise to volitional fatigue at a power output calculated to be either 85% (*n* = 16) or 105% (*n* = 45) of the peak PO achieved during the RI test. A similar approach has been previously described to assess a “true” plateau in the $\dot{\text{V}}$O_2_ response and thus confirm the attainment of a true $\dot{\text{V}}$O_2max_ (Sedgeman et al., 2013). By reducing the relative exercise intensity, the goal in this case was to obtain a longer time to exhaustion, in turn allowing sufficient time for a $\dot{\text{V}}$O_2max_ to be achieved. The highest $\dot{\text{V}}$O_2_ from each test was defined as the greatest consecutive 20-s average during each exhaustive test.

### Measurements

Gas-exchange measurements at the University of Western Ontario were conducted as previously described (Babcock et al., 1994). Briefly, inspired and expired flow rates were measured using a low dead space (90 mL) bidirectional turbine (Alpha Technologies VMM 110) which was calibrated before tests using a syringe of known volume. Inspired and expired gases were sampled continuously (every 20 ms) at the mouth and analyzed for concentrations of O_2_, CO_2_, and nitrogen (N_2_) by mass spectrometry (Perkin Elmer MGA-1100) after calibration with precision-analyzed gas mixtures. Breath-by-breath alveolar gas exchange was calculated using the algorithms of Beaver et al. (1981). Heart rate (HR) was monitored continuously by a three-lead arrangement electrocardiogram using PowerLab (ML132/ML880; ADInstruments, Colorado Springs, CO). Data were recorded using LabChart v4.2 (ADInstruments, Colorado Springs, CO) on a separate computer.

At the University of Verona, breath-by-breath gas exchange and ventilation were continuously measured using a metabolic cart (QuarkB^2^; COSMED, Rome, Italy) as previously described (Keir et al., 2015). The gas analyzers were calibrated before each experiment using a gas mixture of known concentration, and the turbine flowmeter was calibrated using a 3-L syringe (Hans Rudolph, Inc.). HR data were collected using radiotelemetry (SP0180 Polar Transmitter; Polar Electro, Inc., Kempele, Finland) and calculated over the duration of each breath.

### Statistics

All statistics were performed using SPSS version 23 (SPSS, Chicago, IL). Data are presented as means ± *SD*. After ensuring the normal distribution of the data (Shapiro–Wilk test), and equality of variance for the 85% and 105% groups (Levene's test) a three-way repeated-measures ANOVA was used to test the possible influence and interactions of test type (RI test vs. constant load verification phase), age subgroup (Y vs. O) and verification phase intensity (85% vs. 105%) on the highest measures of $\dot{\text{V}}$O_2_ observed during the tests. Significant main effects and interactions were analyzed using the Bonferroni *post-hoc* test. Based on a power calculation, 49 participants were required to identify significant differences between groups for detecting changes larger than our estimated measurement error (i.e., 2 mL·kg^−1^·min^−1^) with a statistical power >0.8. The correlation between the highest $\dot{\text{V}}$O_2_ during the RI test and the constant load verification phase was assessed by Pearson's product moment correlation coefficient. Furthermore, the individual difference between $\dot{\text{V}}$O_2_ observed during the RI test the constant load verification phase was calculated in absolute units (absolute bias in mL·kg^−1^·min^−1^) and as a percent of RI $\dot{\text{V}}$O_2_ (% bias). Bland-Altman plots, followed by a one-sample *z*-test (Bland and Altman, 1986), were used to determine average bias, precision (i.e., standard deviation of the differences between measures) and limits of agreement between the $\dot{\text{V}}$O_2_ measures. The number and percent of subjects in which the RI test either overestimated or underestimated $\dot{\text{V}}$O_2max_ compared to the verification phase by over 2 mL·kg^−1^·min^−1^ (equal to the minimum detectable change as measured in our laboratories for $\dot{\text{V}}$O_2_ measures between 2.1 and 3.5 L·min^−1^) (Keir et al., 2014, 2015) was calculated. For all comparisons, statistical significance was declared when *P* < 0.05.

## Results

**Figure 1:**
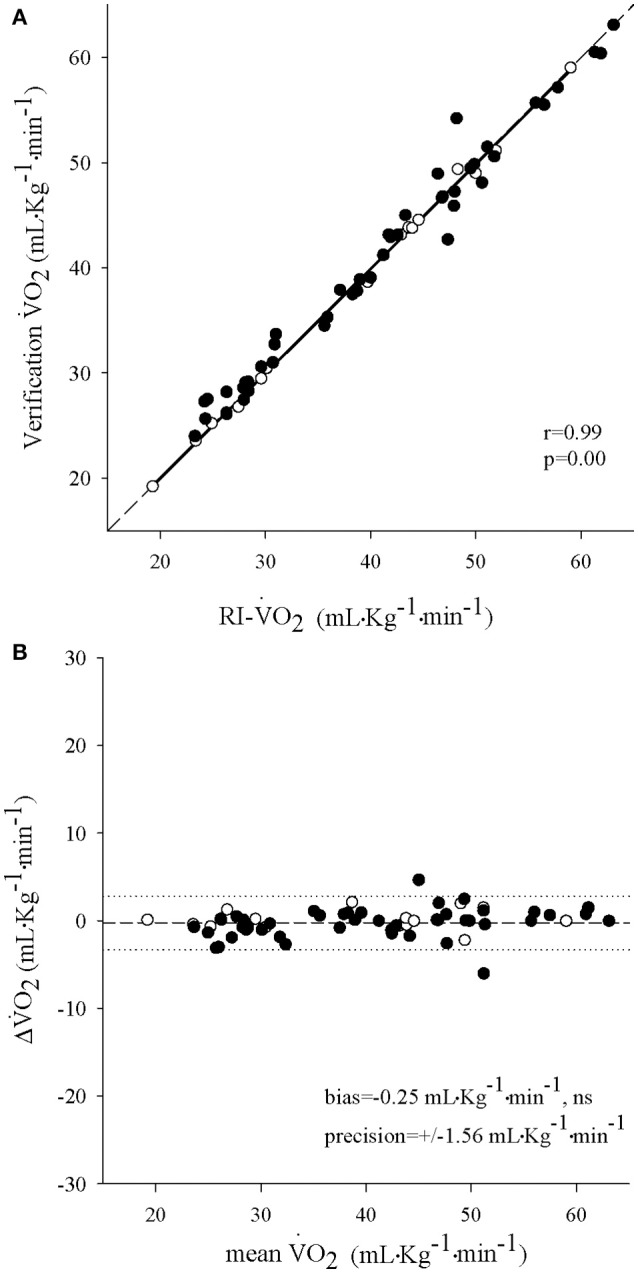
**(A)** The highest individual $\dot{\text{V}}$O_2_ values observed during the verification phase (verification-$\dot{\text{V}}$O_2_) are correlated with the values measured during the ramp incremental test (RI-$\dot{\text{V}}$O_2_). The identity (dashed) and the correlation (solid) lines are displayed along with the correlation coefficient for the entire database. Data of subjects who completed the verification phase at 105 and 85% of maximal power output are displayed as filled (•) and empty circles (°) respectively. **(B)** Individual absolute differences (Δ $\dot{\text{V}}$O_2_) between measures of the highest $\dot{\text{V}}$O_2_ during the RI test and the verification phase are plotted as a function of the average of the two measures. Bias (dashed line) and precision (limits of agreement, dotted lines), for the entire database, are displayed along with the numerical values. Data of subjects who completed the verification phase at 105 and 85% of maximal power output are displayed as filled (•) and empty circles (°) respectively.

The peak PO was significantly lower in O (198 ± 35 W) compared to Y (339 ± 54 W; *P* = 0.86). Time to exhaustion during the RI test was shorter in O (10.2 ± 2.3 min; range 7.9–18.8 min) compared to Y (12.8 ± 2.0 min; range 8.9–18.6 min) (*p* < 0.01). Time to exhaustion during the verification phase was greater when performing constant load exercise at 85% of peak PO (2.5 ± 0.4 min) compared to the constant load exercise performed at 105% of peak PO (1.7 ± 0.4 min) (*p* < 0.01). The average highest $\dot{\text{V}}$O_2_ values during the RI test in the whole group were not significantly different from the $\dot{\text{V}}$O_2_ values observed during the verification phase (Table 1; *P* = 0.33). This response was not affected by age, with no significant differences identified between the RI and verification $\dot{\text{V}}$O_2_ in O or Y (Table 1; *P* = 0.64). Similarly, there were no significant differences in the highest $\dot{\text{V}}$O_2_ values during RI test vs. verification phase at either 85% or 105% of peak PO (*P* = 0.84). The highest $\dot{\text{V}}$O_2_ values observed during both the RI test and the verification phase were highly correlated (*r* = 0.99; *p* < 0.01) (Figure 1A). The Bland-Altman analysis revealed a very small absolute bias (−0.25 mL·kg^−1^·min^−1^), which was not different from 0 (*z* = −1.3), and a precision of ±1.56 mL·kg^−1^·min^−1^ between measures (Figure 1B). Bias ± precision was −0.81 ± 4.14% when expressed as a percent of the RI values. Considering an absolute cut-off error of 2 mL·kg^−1^·min^−1^, the RI test overestimated $\dot{\text{V}}$O_2max_ compared to the verification phase in 4 participants (or ~7% of the group) and underestimated it in 6 participants (or ~10% of the group).

**Table 1:** Highest $\dot{\text{V}}$O_2_ and HR values during the ramp incremental (RI) test and the verification phase for each subgroup.

**Entire data set**	**Total (*n* = 61)**	**Younger (*n* = 30)**	**Older (*n* = 31)**
RI-$\dot{\text{V}}$O_2_ (mL·kg^−1^·min^−1^)	39.8 ± 11.5	48.8 ± 7.7	31.2 ± 7.1
Verification-$\dot{\text{V}}$O_2_ (mL·kg^−1^·min^−1^)	40.1 ± 11.2	48.8 ± 7.5	31.7 ± 6.9
RI-HR (b·min^−1^)	171 ± 20^*^	188 ± 8^*^	153 ± 12^*^
Verification-HR (b·min^−1^)	169 ± 20	185 ± 9	152 ± 14
**Verification 85%**	**Total (*****n*** = **16)**	**Younger (*****n*** = **8)**	**Older (*****n*** = **8)**
RI-$\dot{\text{V}}$O_2_ (mL·kg^−1^·min^−1^)	37.6 ± 11.9	47.2 ± 6.4	28.0 ± 7.1
Verification-$\dot{\text{V}}$O_2_ (mL·kg^−1^·min^−1^)	37.8 ± 11.9	47.6 ± 6.1	28.0 ± 7.0
RI-HR (b·min^−1^)	173 ± 24^*^	192 ± 6^*^	151 ± 14^*^
Verification-HR (b·min^−1^)	170 ± 24	189 ± 7	148 ± 15
**Verification 105%**	**Total (*****n*** = **45)**	**Younger (*****n*** = **22)**	**Older (*****n*** = **23)**
RI-$\dot{\text{V}}$O_2_ (mL·kg^−1^·min^−1^)	40.6 ± 11.4	49.4 ± 8.2	32.3 ± 6.9
Verification-$\dot{\text{V}}$O_2_ (mL·kg^−1^·min^−1^)	40.9 ± 10.9	49.2 ± 8.1	33.0 ± 6.5
RI-HR (b·min^−1^)	170 ± 19^*^	186 ± 8^*^	154 ± 11^*^
Verification-HR (b·min^−1^)	169 ± 19	184 ± 10	153 ± 13

*Average and standard deviation values of $\dot{\text{V}}$O_2_ and HR as derived from the ramp incremental tests (RI) and from the verification trial in the whole group (Total) and in the young and older subgroups. Data from the entire dataset (that combines all tests irrespective of the validation trial intensity) are presented along with the separate averages of the two intensity subgroups*.

* *Significantly different from the verification phase (p < 0.05)*.

Peak HR observed during the RI test (171 ± 20 b·min^−1^) was significantly higher than, and highly correlated with, the HR response observed during the verification phase (169 ± 20 b·min^−1^; *p* < 0.01; *r* = 0.96; *p* < 0.01). Peak HR during both the RI test and the verification phase was significantly lower in O (153 ± 12 b·min^−1^ and 152 ± 14 b·min^−1^, respectively) compared to Y (188 ± 8 b·min^−1^; and 185 ± 9 b·min^−1^, respectively; *p* < 0.01).

## Discussion

This study compared the $\dot{\text{V}}$O_2_ responses from a RI test to the limit of tolerance that was followed by a verification phase to exhaustion performed at intensities of either 85% or 105% of the peak PO observed at the end of the RI protocol, in a large and heterogeneous group of male participants that included older and younger individuals. The study tested whether the verification phase (either below or above the end-RI intensity) would produce a higher $\dot{\text{V}}$O_2_ than the maximal value observed during the RI test and whether such a difference would be affected by verification phase intensity and/or age. The main findings were that: (1) the average highest $\dot{\text{V}}$O_2_ values observed during the verification phases were not significantly higher than those obtained during the RI test; (2) the Bland-Altman analysis revealed a negligible bias (i.e., not different from zero); (3) the RI $\dot{\text{V}}$O_2_ was higher than the measurement error in $\dot{\text{V}}$O_2_ for ~7% and lower for ~10% of the group compared with the verification phase; (4) the difference between maximal $\dot{\text{V}}$O_2_ values associated with the verification phases compared to the RI tests was unaffected by the age of participants and verification phase intensity.

In the context of the unresolved uncertainties surrounding the achievement of a true $\dot{\text{V}}$O_2max_ during a RI test to exhaustion, Poole and Jones (2017) recently proposed that the term $\dot{\text{V}}$O_2peak_ should be avoided and that a verification phase, similar to that proposed by Rossiter et al. (2006) and further studied by others (Astorino et al., 2009; Barker et al., 2011; Sedgeman et al., 2013; Nolan et al., 2014; Sawyer et al., 2015), should become a gold standard, especially in older, frail, and/or unfit populations. The idea that performing a verification phase at a given power output above that observed during the RI test to exhaustion is conceptually appealing as this procedure theoretically confirms that further increases in power output do not result in greater $\dot{\text{V}}$O_2_ values (i.e., an upper limit plateau in the $\dot{\text{V}}$O_2_ response). However, the evidence to support this type of statement appears surprisingly scant, and this idea neglects well-established concepts that show that exercising to the limit of tolerance at any intensity above critical power would result in attainment of $\dot{\text{V}}$O_2max_.

Data from the present study indicated that, in 61 older and younger participants, the average highest $\dot{\text{V}}$O_2_ values observed during the RI tests and the verification phase were similar, irrespective of age, fitness level and verification phase intensity. These findings confirm, in a larger and more heterogeneous population, the findings of others (Rossiter et al., 2006; Astorino et al., 2009; Barker et al., 2011; Sedgeman et al., 2013; Nolan et al., 2014; Sawyer et al., 2015). In addition, our study provides the first data of this type in an older adult population. Furthermore, individual differences between the RI test and the verification phase displayed quantitatively negligible (likely unmeasurable) and not significant bias. The results are similar to those in an athlete sample in whom the $\dot{\text{V}}$O_2_ from RI and verification phase were not different, and within the error of measurement in 24 out of 24 subjects (Weatherwax et al., 2016).

According to the idea originally proposed by Rossiter et al. (2006) and examined by others (Astorino et al., 2009; Barker et al., 2011; Sedgeman et al., 2013; Nolan et al., 2014; Sawyer et al., 2015), and recently endorsed by Poole and Jones (2017), the lack of difference in the highest $\dot{\text{V}}$O_2_ observed in RI vs. the verification phase would provide a proof of a plateau in $\dot{\text{V}}$O_2_, in turn confirming the attainment of a true $\dot{\text{V}}$O_2max_. However, alternate and equally plausible views would be that: (1) the $\dot{\text{V}}$O_2max_ obtained from the RI was “true” in the first place; (2) both the RI and the validation phase suffer from similar limitations (i.e., subjects' inability to endure maximal effort for a long enough time to allow oxidative metabolism to display a maximal functional level). In the first view, the verification phase would not add value beyond performing the RI test alone for providing a true measure of $\dot{\text{V}}$O_2max_. In the second view, Poole and Jones (2017) highlighted that in less experienced participants or those perceived to be less willing to push themselves to their limit of tolerance, the RI model may result in a substantial underestimation of $\dot{\text{V}}$O_2max_ and in such cases, a verification phase might offer an opportunity to highlight such underestimations. The present data set, as well as data from other studies in sedentary (Astorino et al., 2009), recreationally active (Sedgeman et al., 2013; Nolan et al., 2014), and overweight adults (Wood et al., 2010; Sawyer et al., 2015), as well as children (Barker et al., 2011) does not support the idea that a RI test underestimates $\dot{\text{V}}$O_2max_ compared to a verification phase. These findings may in fact support a different interpretation: if $\dot{\text{V}}$O_2max_ values derived from a RI test might underestimate the true $\dot{\text{V}}$O_2max_ in less experienced/fit/healthy individuals, then these same people might not be able or willing to endure a maximal effort during a verification phase. Under these circumstances, the similar $\dot{\text{V}}$O_2_ values observed in both tests might simply represent that participants are equally fatigued, experiencing tired legs or feeling somewhat breathless, but they still remain below their true $\dot{\text{V}}$O_2max_ in both conditions. Thus, the verification phase presenting the same $\dot{\text{V}}$O_2_ values as those observed during the RI test does not imply a plateau that necessarily reflects $\dot{\text{V}}$O_2max_, or a verification of the RI test, but it might rather reflect the lack of its attainment in both tests. In this situation, true achievement of $\dot{\text{V}}$O_2max_ is still uncertain but the “illusion” of having measured a true $\dot{\text{V}}$O_2max_ is created. Given the difficulties associated with determination of a true $\dot{\text{V}}$O_2max_ value, and the limitations being highlighted with the verification phase as a confirmatory test to establish $\dot{\text{V}}$O_2max_, the use of secondary markers of maximal effort become important. Although it has been suggested that secondary criteria might not be valid to provide accurate determination of the attainment of $\dot{\text{V}}$O_2max_ (Midgley et al., 2007; Poole et al., 2008), it could be argued that commonly used criteria such as [La] above 8 mmol·L^−1^, a heart rate response within 10 bpm of the maximal predicted value, an RER higher than 1.10, and an RPE >18, might contribute to feel confidence in the attainment of a true $\dot{\text{V}}$O_2max_ at the end of a RI test.

Perhaps the recommendation for the utilization of a verification phase should recognize that the aim of this approach is not to identify $\dot{\text{V}}$O_2max_ in a majority of participants, but to find the minority of participants who might not achieve it during a RI test. Nevertheless, with a precision of ~1.5 mL·kg^−1^·min^−1^ within the minimum detectable change of 2 mL·kg^−1^·min^−1^, indeed the verification phase revealed very few instances of an underestimation from the RI. Interestingly, the data from the present study showed that the $\dot{\text{V}}$O_2_ values during the RI test similarly over- (i.e., four tests) and underestimated (i.e., six tests) the $\dot{\text{V}}$O_2_ values associated with the verification phase, suggesting major limitations in this verification procedure.

In relation to the effectiveness of the verification phase to reach $\dot{\text{V}}$O_2max_, the relationship between the intensity and the duration of the exercise should be considered. In the present study, the times to exhaustion for the verification phases performed five min after the end of the RI test were ~2.5 and ~1.5 min for exercise performed at 85% and 105% of the peak PO, respectively. Although this time might be considered too short for $\dot{\text{V}}$O_2max_ to develop (especially for more intense and shorter exercise bouts) (Poole and Jones, 2012), the fact that the verification phases were performed shortly after the end of the RI test and that the system was “primed” (De Roia et al., 2012) might have contributed to the achievement of a $\dot{\text{V}}$O_2_ response that was as high as that seen toward the end of the RI test. While some papers have used supramaximal intensities of exercise for the verification phases in order to establish a “plateau response” despite an increase in PO beyond that observed during the RI test (Astorino et al., 2009; Barker et al., 2011; Nolan et al., 2014), Sedgeman et al. (2013) compared a verification phase performed at 105% of the peak PO during the RI test to a verification phase performed at a PO equivalent to that occurring two stages before the end of a graded test (i.e., ~54 W lower PO or ~82% of peak PO), subsequent to 3 min recovery after the end of the RI test. As found for the present data using 85% or 105% of the end-RI work rate, Sedgeman et al. (2013) also showed the $\dot{\text{V}}$O_2_ values were not different between the RI test and the verification phase either with the sub-maximal (~2.2 min duration) or the supramaximal (~1.3 min duration) verification. These authors suggested that a sub-maximal verification phase might be beneficial as more plateau responses were observed during this type of verification. This interpretation is not surprising; exercising within the very heavy intensity domain (such as in Sedgeman et al. and the present studies) is compatible with a longer exercise duration, in turn allowing enough time for the slow component of $\dot{\text{V}}$O_2_ to develop and for $\dot{\text{V}}$O_2max_ to be achieved. On the contrary, exercising in the severe intensity domain (e.g., 105% of peak PO) might pose some extra challenges for confirmation of a true $\dot{\text{V}}$O_2max_ as a plateau response is less likely to occur and, although not the case in the present study and in that of Sedgeman et al., could theoretically result in muscle fatigue before the $\dot{\text{V}}$O_2_ response can be fully developed and $\dot{\text{V}}$O_2max_ expressed (Poole and Jones, 2012). This is an important aspect to consider as the duration of recovery period before the verification phase and/or the characteristics of the ramp during the incremental test to exhaustion (i.e., steeper ramps will result in higher peak POs compared to less steep ramps) might determine whether or not the highest possible $\dot{\text{V}}$O_2_ is achieved when the intensity of the verification phase is supramaximal.

In the present study, the verification phase at 85% and 105% of peak PO were collected in two different laboratories, which might represent a possible limitation. However, the equipment used in both laboratories was comparably accurate and precise and the experimental procedures were consistent between laboratories and clearly described to allow replication. Furthermore, since the analysis compares $\dot{\text{V}}$O_2_ values obtained with the RI test and verification phase within each individual, whatever systematic bias in $\dot{\text{V}}$O_2_ measures might exist between laboratories would affect both data-points in the same way. As for the vast majority of the studies that have described the use of a verification phase, the population tested in the present study is limited to male participants. It was important to verify the usefulness of the verification phase in the same population in which the verification phase has been proposed; however, the absence of female participants in this study is an issue that, although not expected to change the outcome observed in the present investigation, might need to be addressed in future studies.

In conclusion, the accurate identification of $\dot{\text{V}}$O_2max_ remains an elusive issue and there is a clear need for developing reliable criteria to objectively evaluate the achievement of a true maximal response. In this context, the proposal by Poole and Jones (2017) to use the verification phase for true $\dot{\text{V}}$O_2max_ determination appeared a very promising approach to overcome the absence of an objective plateau in $\dot{\text{V}}$O_2_. However, data presented in this study indicate that a verification phase performed subsequent to the end of a RI test does not provide a convincing confirmation that a true $\dot{\text{V}}$O_2max_ response has been achieved. Therefore, the recommendation that a verification phase should become the gold standard procedure for $\dot{\text{V}}$O_2max_ determination, although initially appealing, is not supported. As such, the verification phase should not be accepted as a gold standard, and given the present analysis indicating that in most cases the $\dot{\text{V}}$O_2_ during the RI test is as high as that of the verification phase, or that the highest $\dot{\text{V}}$O_2_ in the few cases observed on either the RI test or the verification phase is within the error of the measurement, further efforts to endorse the verification phase to confirm $\dot{\text{V}}$O_2max_ do not seem to be a tenable direction for future research.

## Author contributions

JM, SP, and DP: contributed to the conception of the work, analysis, and interpretation of data; JM, SP, and DP: contributed to the drafting and revisions of the manuscript; JM, SP, and DP: approved the final version of this manuscript; JM, SP, and DP: agree to be accountable for all aspects of the work in ensuring that questions related to the accuracy or integrity of any part of the work are appropriately investigated and resolved.

### Conflict of interest statement

The authors declare that the research was conducted in the absence of any commercial or financial relationships that could be construed as a potential conflict of interest.
